# Resolutions on the Death of Dr. George H. Cushing

**Published:** 1900-10-15

**Authors:** Louis Ottofy, W. C. Skidmore, Lloyd R. Hawley

**Affiliations:** Committee. Manila, P. I.; Committee. Manila, P. I.; Committee. Manila, P. I.


### RESOLUTIONS ON THE DEATH OF DR. GEORGE H. CUSHING.

The intelligence having been received by the members of the dental profession of Manila that the spirit of the venerable Dr. Geo. H. Cushing had passed away, and recognizing the great loss to the dental profession by reason of his death, and furthermore feeling that the beneficient influence of his long and useful career as a practitioner and teacher are operative in even this distant land, the members of the Manila Dental Society have come together to do honor to his memory, and have adopted the following resolution :

WHEREAS, The Almighty in His infinite wisdom, has spared the life of our friend and teacher, until, though passed the alloted life of man, he was still in possession of the strength and power of mind to be serviceable as a teacher; and,

WHEREAS, The world is better by reason of his busy life, the dental profession richer in her store of knowledge and the hearts of his friends sorrowful in the consciousness of his departure from among them; now, therefore, be it

*Resolved*, That the members of this Society, by this action, add their tribute to his worth, express their sincere and heartfelt sympathy to his bereaved family and to the dental profession of the United States and that this resolution be entered of record, a copy be sent to the family of the deceased and to the dental journals for publication.

LOUIS OTTOFY,<br>

W. C. SKIDMORE, } *Committee.*<br>

LLOYD R. HAWLEY,

MANILA, P. I., August 6, 1900.

